# Metabolome and Transcriptome Analyses of Cucurbitacin Biosynthesis in *Luffa* (*Luffa acutangula*)

**DOI:** 10.3389/fpls.2022.886870

**Published:** 2022-06-07

**Authors:** Gangjun Zhao, Meng Wang, Caixia Luo, Junxing Li, Hao Gong, Xiaoming Zheng, Xiaoxi Liu, Jianning Luo, Haibin Wu

**Affiliations:** ^1^Guangdong Key Laboratory for New Technology Research of Vegetables, Vegetable Research Institute, Guangdong Academy of Agricultural Sciences, Guangzhou, China; ^2^Guangdong Laboratory for Lingnan Modern Agriculture, Guangzhou, China

**Keywords:** *Luffa*, Cucurbitaceae, metabolome, cucurbitacin, abiotic stress

## Abstract

Cucurbitacins are extremely bitter compounds mainly present in Cucurbitaceae, where *Luffa* belongs. However, there is no comprehensive analysis of cucurbitacin biosynthesis in *Luffa* fruit. Therefore, this study analyzed bitter (WM709) and non-bitter (S1174) genotypes of *Luffa* to reveal the underlying mechanism of cucurbitacin biosynthesis by integrating metabolome and transcriptome analyses. A total of 422 metabolites were detected, including vitamins, essential amino acids, antioxidants, and antitumor substances. Of these, 131 metabolites showed significant differences between bitter (WM709) and non-bitter (S1174) *Luffa* fruits. The levels of isocucurbitacin B, cucurbitacin D, 23,24-dihydro cucurbitacin E, cucurbitacin F were significantly higher in bitter than in non-bitter *Luffa*. Transcriptome analysis showed that *Bi*, cytochromes P450s (CYP450s), and acyltransferase (ACT) of the cucurbitacin biosynthesis pathway, were significantly up-regulated. Moreover, drought stress and abscisic acid (ABA) activated genes of the cucurbitacin biosynthesis pathway. Furthermore, dual-luciferase reporter and yeast one-hybrid assays demonstrated that ABA-response element binding factor 1 (AREB1) binds to the *Bi* promoter to activate *Bi* expression. Comparative analysis of the *Luffa* and cucumber genomes showed that *Bi*, CYP450s, and *ACT* are located in the conserved syntenic loci, and formed a cucurbitacin biosynthesis cluster. This study provides important insights into major genes and metabolites of the cucurbitacin biosynthetic pathway, deepening the understanding of regulatory mechanisms of cucurbitacin biosynthesis in *Luffa*.

## Introduction

*Luffa* [*Luffa acutangula* (L.) Roxb.] is a member of the Cucurbitaceae family, with a long history of cultivation in tropical and subtropical regions (Kalloo, 1993; Wu et al., 2016). The *Luffa* genus has nine species, but only *L. acutangula* Roxb. and *L. cylindrica* Roem. are domesticated (Walters, 1989; Gautam, 2017; Wu et al., 2020). The crop is highly nutritious, rich in vitamins (A, B, and C), minerals (P, K, Ca, Na, S, Fe, Zn, and Mn), and amino acids (aspartic acid, glutamic acid, alanine, cysteine, and tyrosine; Swetha and Muthukumar, 2016; Shendge and Belemkar, 2018; Zhang et al., 2020). Moreover, *Luffa* is an important medicinal plant. *Luffa* has anti-HIV-1, anticancer, hepatoprotective, antidiabetic, antiulcer, antioxidant, fungistatic, analgesic, larvicidal, and immunomodulatory activities (Ng et al., 2011; Kuppast and Mankani, 2012; Shendge and Belemkar, 2018). Recent research also shows that compounds isolated from *Luffa* spp. function as anti-SARS-CoV-2 main protease (Cao et al., 2021).

Cucurbitacins are oxygenated tetracyclic triterpenoids divided into 12 categories of extremely bitter compounds, mainly in the Cucurbitaceae family (Vieira et al., 2019; Ma et al., 2022). Many cucurbitacins have antitumor activities, but the activities are different because of their varied structural characteristics (Iwanski et al., 2010; Jevtić et al., 2016; Yang et al., 2020). Various Cucurbitaceae crops have different cucurbitacins, namely Cucurbitacins B (CuB) in melon, CuC in cucumber, CuE and CuI in bottle gourd, and CuE in watermelon (Shang et al., 2014; Mashilo et al., 2018; Gong et al., 2021; Xu et al., 2022).

Typically, cucurbitacin synthesis starts with cyclization of 2,3-oxidosqualene to cucurbitadienol in Cucurbitaceae plants, catalyzed by oxidosqualene cyclases (OSCs or Bi; Shang et al., 2014; Zhou et al., 2016). However, a basic helix–loop–helix (bHLH) transcription factor in bitter fruit (Bt) regulates Bi (Shang et al., 2014), while CYP450s oxidize cucurbitadienol, which is further acetylated by ACT to produce various cucurbitacin (Shibuya et al., 2004; Shang et al., 2014; Zhou et al., 2016). Comparative analyses of cucumber, melon, and watermelon genomes showed that *Bi*, CYP450s, and ACT are located in the conserved syntenic loci, and formed a cucurbitacin cluster with biosynthesis-related functionality (Shang et al., 2014; Zhou et al., 2016; Xu et al., 2022). In cucumber, the UDP-glucosyltransferase UGT73AM3 regulates glucosylation of bitter triterpenoid (Zhong et al., 2017; Yang et al., 2019).

Biotic and abiotic stress (i.e., diseases, insect pests, salt, heat, and drought stress) induce cucurbitacin biosynthesis in plants (Shang et al., 2014; Yoshida et al., 2014; Pan et al., 2022). The plant hormone ABA, produced in abiotic-stressed plants, is critical for mediating plant responses to stressful environmental conditions (Yoshida et al., 2014; Li et al., 2021b). Moreover, ABA-response element binding factors (AREBs) are activated in an ABA-dependent pathway and bind to the ABA-responsive elements (ABREs) in the promoters of downstream genes, thus, activating downstream genes (Yoshida et al., 2014; Li et al., 2021a). AREB1 regulates the ABRE-dependent ABA signaling that enhances abiotic tolerance in *Arabidopsis* (Uno et al., 2000; Furihata et al., 2006). Moreover, AREB1 has similar functions in many plants, including rice, maize, tomato, and *Populus trichocarpa* (Uno et al., 2000; Schütze et al., 2008; Orellana et al., 2010; Yoshida et al., 2010; Li et al., 2018). In cucumber, cucurbitacin synthesis-related genes *Bt*, *Bi*, CYP450s, and *ACT*, were up-regulated under drought, cold, or ABA treatments, promoting cucurbitacin C biosynthesis (Shang et al., 2014).

Nevertheless, there is no comprehensive report on cucurbitacin biosynthesis combining the metabolome and transcriptome of *Luffa* fruits. Besides, the relationship between cucurbitacin biosynthesis and the ABA signaling pathway remains unknown. Therefore, this study combined transcriptome and metabolome analyses to comprehensively analyze the characteristics of metabolic components and molecular mechanisms underlying cucurbitacin biosynthesis in bitter *Luffa*. Furthermore, the study established a direct relationship between cucurbitacin synthesis and abiotic stress.

## Materials and Methods

### Plant Materials

S1174, a *Luffa* inbred line with non-bitter fruit, and WM709, bitter fruit germplasm that has been selfed for multiple generations were used in this study. Both varieties were cultivated in an open field in Baiyun experimental base under normal field conditions, including irrigation, fertilization, pest, and disease control. In brief, organic matter (3,000 kg/ha) and NPK (500 kg/ha) fertilizers were added to the soil before planting, plus two to three additions of NPK compound fertilizer (50 kg/ha) during the growing season. The plants were irrigated at 4–5-day intervals. Fruit flies were controlled by spraying plants with 1.8% avermectin EC. Downy mildew was controlled with 75% chlorothalonil WP 600 solution, while powdery mildew was controlled with 20% triadimefon 2000 solution. Finally, the fruits were collected 10 days after pollination, cut into three sections, wrapped with tin foil, and immediately frozen in liquid nitrogen. This study had three replicates with five fruits per replicate.

### Metabolomic Analysis

The Wuhan Metware Biotechnology Co., Ltd.^1^ extracted, detected, and quantified the metabolites following standard procedures (Chen et al., 2013; Wang et al., 2019). Briefly, the metabolites in freeze-dried fruits were extracted overnight in 70% methanol at 4°C. Afterward, the samples were centrifuged at 10,000 *g* for 10 min, and the extracts were absorbed and filtered for further analysis on an LC-ESI-MS/MS system (UPLC, Shim-pack UFLC SHIMADZU CBM30A system, Kyoto, Japan; MS, Applied Biosystems 4500 Q TRAP, Applied Biosystems, MA, United States).

All detected metabolites were annotated on the MetWare database and quantified using the multiple reaction monitoring (MRM) method. Metabolite data analysis was conducted using Analyst 1.6.1 software. An orthogonal projection to latent structures discriminant analysis (OPLS-DA) model was established using multiple supervision methods. The relative importance of each metabolite to the OPLS-DA model was checked using the variable importance in projection (VIP) value. Metabolites with significantly different contents were determined using the VIP ≥ 1 and |log2(fold change) | ≥ 1 thresholds. After that, the differential metabolites were mapped to the Kyoto Encyclopedia of Genes and Genomes (KEGG) database (Kanehisa et al., 2007) to identify significantly enriched pathways.

### Transcriptome Analysis

Total RNA was extracted from six samples (three replicates each of S1174 and WM709) using the TRIZOL reagent (TaKaRa, Kusatsu-Shiga, Japan) following the manufacturer’s instructions. The RNA quality was confirmed using the Agilent 2100 Bioanalyzer (Agilent, CA, United States). The cDNA libraries were sequenced on the Illumina Hiseq2500 platform following the 150 bp paired-end protocol, and reads were processed using Trimmomatic (Bolger et al., 2014). Clean reads were assembled and aligned to the S1174 reference genome (unpublished) using Hisat2 (Kim et al., 2019). DESeq2 (Love et al., 2014) was used to identify differentially expressed genes (DEGs) using the |log2(fold change)| ≥ 1 and padj <0.05 threshold. The functions of DEGs were determined using the Gene Ontology (GO; The Gene Ontology Consortium, 2018) and KEGG databases (Kanehisa et al., 2007).

### Synteny Analysis

For synteny analysis, the cucumber genomic FASTA and feature files (.gff files) were downloaded from the CuGenDB^2^ and used as input files in the one-step MCScanX (Wang et al., 2012) algorithm of TBTools (Chen et al., 2020) toolkit.

### Drought Stress and ABA Treatment

For drought stress, *Luffa* was grown at 26 ± 2°C with a 16/8 h light/dark cycle in a climate-controlled room. The 12-day-old *Luffa* seedlings were not watered, while control plants were watered at 2–3-day intervals to keep the soil moist. The leaves began to wilt on the fourth day and were sampled the same day. For the ABA treatment, 12-day-old seedlings were sprayed with a 100 μM ABA solution, while control plants were sprayed with mock solution (water). Leaves were sampled 6 h after treatment (ABA or mock). All the samples were frozen in liquid nitrogen for qRT-PCR analyses. One biological replicate consisted of five plant samples, and three biological replicates were analyzed.

### Quantitative Real-Time PCR

Total RNA was isolated from the test samples and used to generate cDNA. The qRT-PCR analysis was performed in the Bio-Rad CFX96 machine (Bio-Rad Laboratories, CA, United States) using the SYBR Premix Ex Taq Kit (TaKaRa, Kusatsu-Shiga, Japan). The 2^−△△CT^ method was used to calculate the fold change of gene expression, and the data were log2 transformed before analysis (Liu et al., 2017). The gene-specific primers used are listed in Supplementary Table S1, and 18s rRNA was the internal reference gene.

### Dual-Luciferase Reporter Assays

Here, full-length open reading frames (ORFs) of *AtAREB1* and *LaAREB1* were amplified from the *Arabidopsis thaliana* and S1174 cDNA and cloned into pGreenII 62-SK (Hellens et al., 2005). The -2000--1201 region of *LaBi* promoter fragment was ligated into pGreen II 0800-LUC (Hellens et al., 2005). *Arabidopsis* protoplasts were prepared and transformed with the described vectors, following a previous protocol (Yoo et al., 2007). The firefly luciferase (LUC) and renilla luciferase (REN) activity assays were performed *via* the dual-luciferase reporter assay system (Promega, WI, United States). The promoter activity was shown as the ratio of LUC to REN.

### Yeast One-Hybrid Assays

Full-length ORFs of *AtAREB1* and *LaAREB1* were amplified from the *A. thaliana*, S1174 cDNA, and cloned into pGADT7. The -2000--1201 region of the *LaBi* promoter fragment was ligated into pHis2. Yeast one-hybrid (Y1H) analysis (Clontech Laboratories Inc., CA, United States) was performed to examine AtAREB1 and LaAREB1 binding to the *LaBi* promoter.

## Results

### Identification of Metabolites in the Fruits of Two *Luffa* Varieties

**Figure 1:**
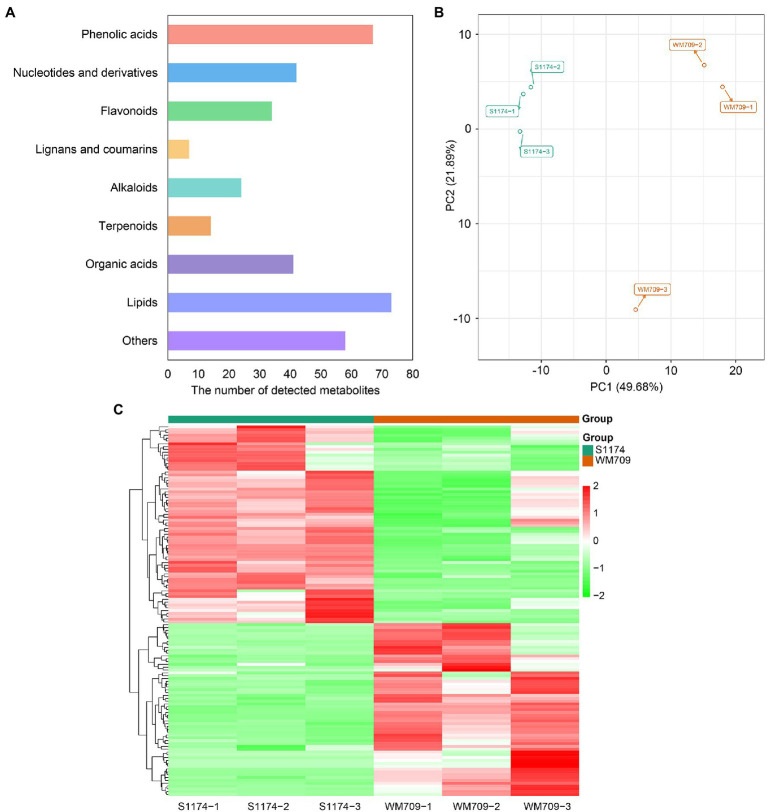
Preliminary analysis of metabolomics data. **(A)** Classification of detected metabolites. **(B)** Principal component analysis (PCA) of detected metabolites. **(C)** Clustered heatmap based on the metabolites between S1174 and WM709.

Metabolome analysis was performed to unravel the metabolite differences between S1174 and WM709 varieties of *Luffa*. The total ions current (TIC) and multiple reactions monitoring (MRM) profiles detected 422 metabolites, including 73 lipids, 67 phenolic acids, 62 amino acids and derivatives, 58 other compounds, 42 nucleotides and derivatives, 41 organic acids, 34 flavonoids, 24 alkaloids, 14 terpenoids and seven lignans and coumarins in the two varieties (Figure 1A; Supplementary Table S2). A principal component analysis (PCA) of the detected metabolites showed that the first two principal components explain 49.68% (PC1) and 21.89% (PC2) of the samples’ variance, respectively (Figure 1B). Besides, a heatmap analysis clearly distinguished the metabolites in the two varieties (Figure 1C). Thus, the six samples were separated into two groups according to their OPLS-DA (Supplementary Figure S1A). R2X, R2Y, and Q2 were 0.728, 0.999, and 0.895, respectively (Supplementary Figure S1B). The R2 and Q2 values were close to 1.0, suggesting a satisfactory model with a reliable predictive ability.

### Functional Analysis of Metabolites

The differentially accumulated metabolites (DAMs) between the two *Luffa* varieties were determined based on the variable importance in the projection (VIP) ≥ 1 and a | Log2Fold Change | ≥ 1. Essentially, 131 metabolites were significantly different between the varieties, with 61 up-regulated and 70 down-regulated (Supplementary Table S3). The DAMs included 26 lipids, 25 phenolic acids, 24 nucleotides and derivatives, 11 terpenoids, 11 flavonoids, nine organic acids, nine other compounds, six amino acids and derivatives, five alkaloids, and five lignans and coumarins.

**Figure 2:**
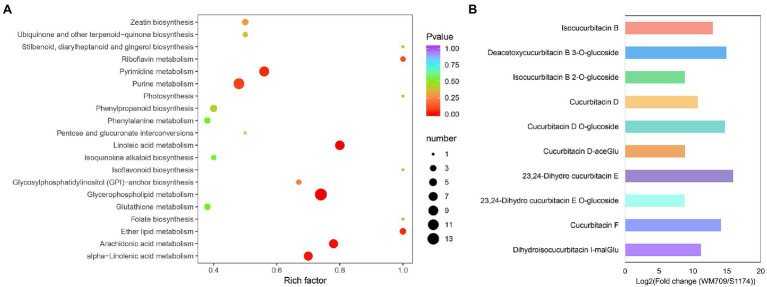
Analyses of differentially accumulated metabolites (DAMs) between WM709 and S1174. **(A)** The top 20 enriched Kyoto Encyclopedia of Genes and Genomes (KEGG) pathways. **(B)** Detected cucurbitacins in WM709.

KEGG pathway analysis for the DAMs showed that they were enriched in metabolism, ABC transporters, and aminoacyl−tRNA biosynthesis pathways (Figure 2A; Supplementary Table S4). The top five significantly enriched pathways were glycerophospholipid (ko00564), alpha−Linolenic acid (ko00592), purine (ko00230), pyrimidine (ko00240), and linoleic acid metabolism (ko00591). In addition, the pathway of ubiquinone and terpenoid-quinone biosynthesis metabolite (ko00130) was significantly enriched. In bitter *Luffa* S1174, the high level of metabolites enriched purine metabolism (ko00230), pyrimidine metabolism (ko00240), and ABC transporters (ko02010; Supplementary Figure S2).

Moreover, the contents of terpenoids, including cucurbitacin D, cucurbitacin F, 23,24-dihydro cucurbitacin E, isocucurbitacin B, deacetoxycucurbitacin B 3-O-glucoside, cucurbitacin D O-glucoside, isocucurbitacin B 2-O-glucoside, cucurbitacin D-aceGlu, 23,24-dihydro cucurbitacin E O-glucoside, and dihydroisocucurbitacin I-malGlu, were significantly higher in bitter *Luffa* than in non-bitter *Luffa* (Figure 2B; Supplementary Table S4).

### Differentially Expressed Genes in the Fruits of Two *Luffa* Varieties

The transcriptome sequence of S1174 and WM709 generated 54.20 Gb of clean data, with >94.19% of the bases scoring Q30 (Supplementary Table S5). Over 95.42% of clean reads were mapped to the reference genome, generating >92.76% uniquely mapped sequences (Supplementary Table S6). The correlation coefficients (Supplementary Figure S3A) and PCA (Supplementary Figure S3B) indicated expression pattern similarities between replicate samples. Finally, 2,634 DEGs were identified, including 1,455 up-regulated and 1,179 down-regulated genes (Supplementary Table S7). The transcriptome analysis identified 222 DEGs (98 up-regulated and 124 down-regulated) belonging to 37 families of transcription factors (TFs) between WM709 and S1174 (Supplementary Figure S4). The MYB, AP2/ERF, bHLH, C2C2, and NAC families were the top five TF among the DEGs (Supplementary Figure S4).

### Functional Analysis of DEGs

**Figure 3:**
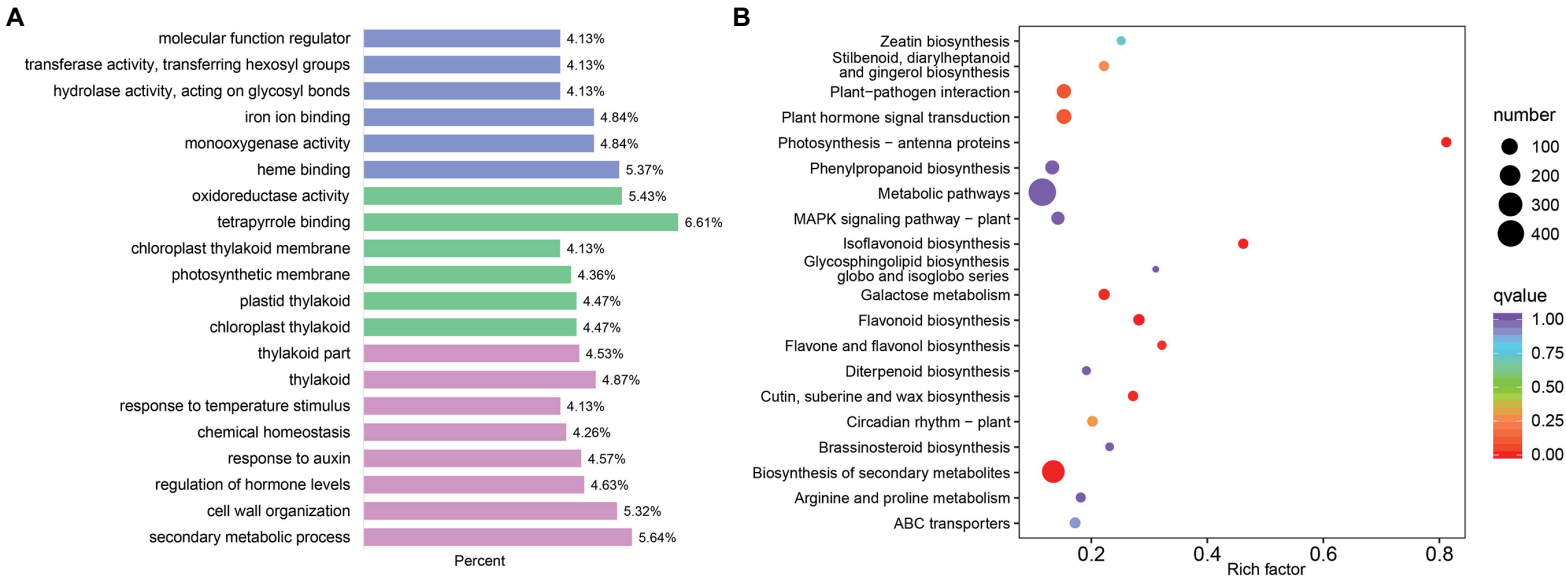
Differentially expressed genes between WM709 and S1174. **(A)** Top 20 of Gene Ontology (GO) annotations of differentially expressed genes (DEGs). Blue bar, biological process; Green bar, molecular function; and Pink bar, cellular components. **(B)** Top 20 enriched GO annotations using the DEGs.

The GO enrichment analysis identified 135 significantly enriched GO terms (Figure 3A; Supplementary Table S8). The significantly enriched GO terms included 82 biological processes, such as secondary metabolic process (GO:0019748), flavonoid biosynthetic process (GO:0009813), and photosynthesis (GO:0015979). The 38 significantly enriched molecular functions included galactosidase activity (GO:0015925), glucosidase activity (GO:0015926), chlorophyll binding (GO:0016168), and UDP-glycosyltransferase activity (GO:0008194). Fifteen GO terms were significantly enriched in the cellular component, including thylakoids (GO:0009579) and photosystem (GO:0009521).

Besides, the KEGG enrichment analysis showed that the DEGs were mainly involved in metabolic pathways (ko01100), biosynthesis of secondary metabolites (ko01110), photosynthesis-antenna proteins (ko00196), flavonoid biosynthesis (ko00941), flavone and flavanol biosynthesis (ko00944; Figure 3B; Supplementary Table S9). Additionally, terpenoid synthesis, including terpenoid backbone biosynthesis (ko00900), monoterpenoid biosynthesis (ko00902), diterpenoid biosynthesis (ko00904), sesquiterpenoid and triterpenoid biosynthesis (ko00909), ubiquinone, and another terpenoid-quinone biosynthesis (ko00130) were significantly enriched.

### Expression of Cucurbitacin Biosynthetic Pathway Genes

**Figure 4:**
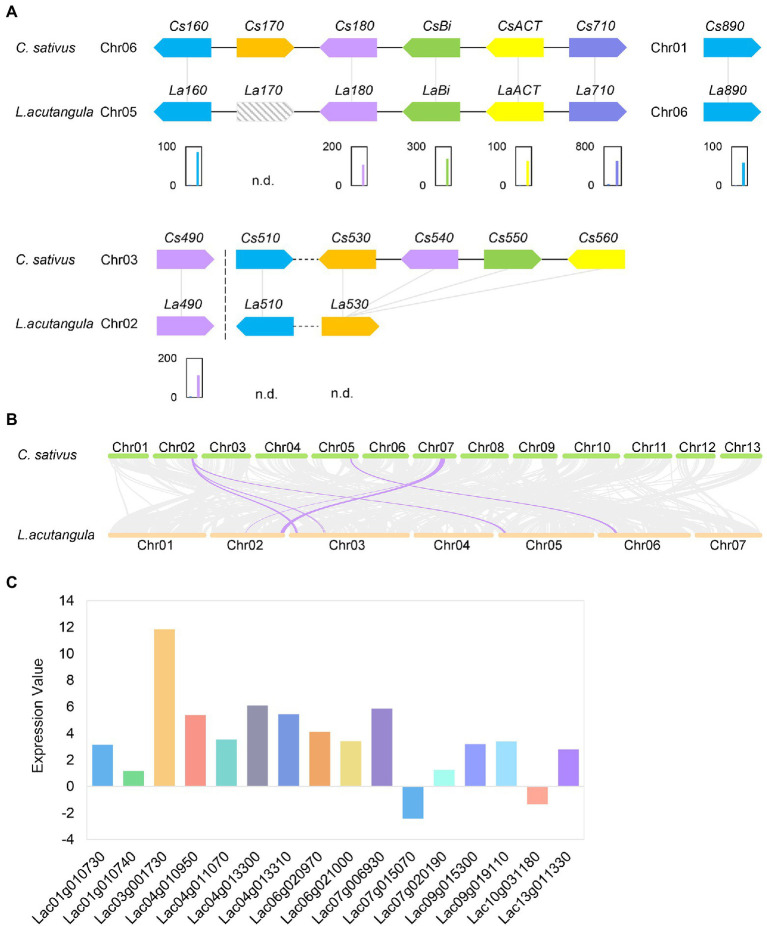
Cucurbitacin biosynthesis genes. **(A)** The distribution and expression of cucurbitacin biosynthesis genes are shown by the fragments per kilobase of transcript per million (FPKM) values. Left bar, S1174 and Right bar, WM709. n.d., not detected. **(B)** Synteny blocks between cucumber and *Luffa*. **(C)** Expression analysis of *UGT*s between WM709 and S1174 *Luffa* varieties. The height of the columns represents the log2 [fold change (WM709/S1174)].

Considering the cucurbitacin biosynthesis and regulatory pathway in cucumber, we used the amino acid sequences of cucumber genes as the query. Thus, a BLASTP search was performed for *Bt*, *Bi*, *ACT*, CYP450s, and UDP-Glucosyltransferases (UGTs) homologous genes in *Luffa* (Supplementary Table S10). The results showed that both S1174 and WM709 had undetectable levels of *LaBt* (*Lac02g016030*; Supplementary Table S7). The *LaBi* (*Lac05g013070*) gene, homologous to cucumber *Bi*, encodes an oxidosqualene cyclase (OSC) responsible for the first committed step in the generation of cucurbitane skeleton, catalyzing 2,3-oxidosqualene to cucurbitadienol, was up-regulated in WM709 than S1174 (Figure 4A; Supplementary Table S7). Besides, five *CYP450s* (*La160*, *La180*, *La710*, *La890*, and *La490*) and *ACT* gene were significantly up-regulated (Figure 4B; Supplementary Table S7). The expression of *La170* gene (located in chromosome 5) was not detected in S1174 and WM709 *Luffa* varieties (Supplementary Tables S7 and S10). Notably, *Cs530*, *Cs540*, *Cs550*, and *Cs560* were homologous to *La530* (Figure 4A). The expression levels of *La530* and *La510* were undetectable in S1174 and WM709 (Figure 4A; Supplementary Table S7). A Bi cluster containing *LaBi*, *LaACT*, and three *CYP450s* (Figure 4A) was in the synteny block between chromosome 6 of cucumber and chromosome 5 of *Luffa* (Figure 4B). Besides, the metabolome levels of five cucurbitacin glucosides increased in bitter *Luffa* WM709 compared to non-bitter *Luffa* S1174 (Figure 2B; Supplementary Table S4). Sixty-six UGT genes were identified in the *Luffa* genome, including 16 significantly regulated genes (14 up-regulated and two down-regulated; Figure 4C; Supplementary Table S7).

### Drought Stress and ABA Treatment Activate Cucurbitacin Biosynthesis Genes

**Figure 5:**
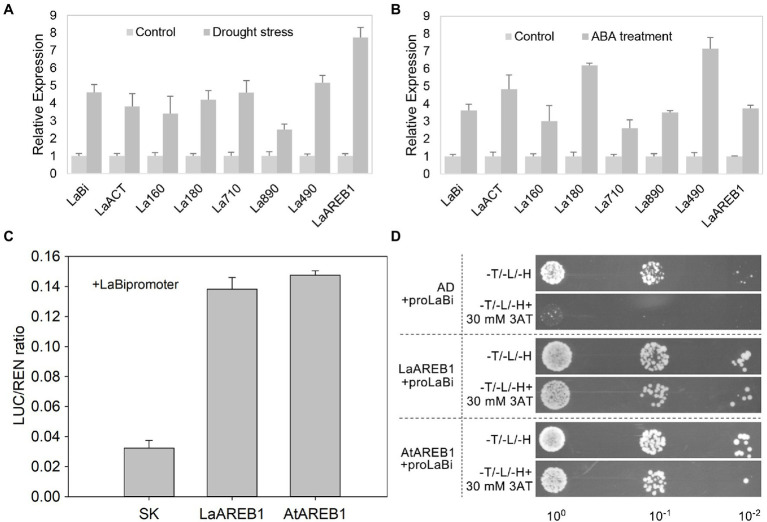
Stress-activated cucurbitacin biosynthesis genes. **(A)** Expression of *Bi*, *ACT*, and *CYP450s* after drought stress. **(B)** Expression of *Bi*, *ACT*, and *CYP450s* after ABA treatment. **(C)** Yeast one-hybrid analysis of the interactions between the LaAREB1 and AtAREB1 proteins and the *LaBi* promoters. **(D)** Dual-Luciferase reporter assays show the interactions between LaAREB1 and AtAREB1 proteins and the *LaBi* promoters.

*Luffa* seedlings were subjected to drought stress and ABA treatment to verify whether stress activates cucurbitacin genes. The results showed that drought stress and ABA treatment significantly increased the expression of *Bi*, *ACT*, and *CYP450s* (Figures 5A,B). Indeed, the *Bi* promoters in *Luffa* contained ABREs (Supplementary Table S11). AREB1 might directly regulate *LaBi* expression; therefore, subsequent dual-luciferase reporter assays were performed to determine whether *LaBi* is a direct target gene of the LaAREB1 protein. The results showed that co-expressing 35S::LaAREB1 and LaBipro::LUC drastically increased the luminescence intensity, indicating that LaAREB1 binds to the *LaBi* promoter. These results were further confirmed by co-expressing 35S::AtAREB1 and LaBipro::LUC (Figure 5C), and performing yeast one-hybrid assays. As indicated in Figure 5D, co-transformed yeast strains harboring pGADT7-LaAREB1 and pHIS2-LaBi exhibited normal growth in a selective medium, confirmed by pGADT7-AtAREB1. These results show that AREB1 positively regulates *LaBi* expression by directly binding to its promoter.

## Discussion

### *Luffa* Is an Important Dietary and Medicinal Plant

*Luffa*, a common vegetable in the daily diet, contains substantial amounts of phytochemicals (Zhang et al., 2020). The fruit is rich in dietary fiber, essential amino acids, vitamins, minerals, polyphenols, flavonoids, saponin, triterpenoids, oleanolic acid, carotenoids, and other nutrients (Zhang et al., 2021). In this study, we conducted a comprehensive targeted metabolomic analysis to determine the metabolite compositions of two *Luffa* varieties. Notably, human essential amino acids and their derivatives, including L- (+)-Lysine, L- (−)-Tyrosine, L-Phenylalanine, L-Tryptophan, L-Valine, and L-Leucine. Nicotinic acid (Vitamin B_3_), pyridoxine (Vitamin B_7_), riboflavin (Vitamin B_2_), and D-pantothenic acid (Vitamin B_5_) were detected in the fruits of the two *Luffa* varieties. Besides, the *Luffa* fruits had γ-Vitamins such as linolenic acid and their derivatives, punic acid, and α-essential unsaturated fatty acids, including linolenic, eicosenoic, and octadecatetraenoic acid. The fruits are also rich in saccharides, including sucrose, glucose, mannose, and galactose. As *Luffa* is rich in nutrients, it has become an important dietary vegetable.

Several studies have demonstrated that consuming *Luffa* considerably supplies antioxidant, anti-inflammation, anticancer, and anti-bacterial constituents to the human body (Du et al., 2006). In this study, abundant methyl ferrate, syringic acid, 1-O-[(E)-caffeoyl], β-D-glucopyranose, punic acid, diosmetin, cymaroside, cucurbitacin B, D, E, I, and their glucosides were detected in the *Luffa* fruits. These compounds have important roles in antimicrobial, antioxidant, and anti-inflammatory activities and can inhibit cancer proliferation (Du et al., 2006; Patel et al., 2013; Winkler-Moser et al., 2015; Aruna et al., 2016; Srinivasulu et al., 2018; Sikander et al., 2019). Therefore, *Luffa* fruits can be considered an essential dietary food and medicinal plant (Partap et al., 2012).

### Cucurbitacin Biosynthesis Pathway in *Luffa*

The Bi cyclization of 2,3-oxidosqualene to cucurbitadienol initiates cucurbitacin biosynthesis. Bi is considered the first key pathway-specific enzyme for cucurbitacin biosynthesis in cucurbits (Shibuya et al., 2004; Shang et al., 2014). *CYP450* genes are often part of gene clusters in specialized metabolism, especially for mono-, di-, and tri-terpenoid synthesis in cucurbits and tomato (Dar et al., 2021). CYP450s tailor the cucurbitacin core skeleton and key structural variations among cucurbitacins B, C, and E in Cucurbitaceae (Zhou et al., 2016). Moreover, ACT acetylates cucurbitadienol to form various cucurbitacin (Shang et al., 2014; Zhou et al., 2016). Bt activates Bi and regulates CuC biosynthesis in cucumber fruit (Shang et al., 2014). In cucumber, melon, and watermelon fruits, *Bt*, *Bi*, *CYP450s*, and *ACT* form gene clusters to regulate cucurbitacin synthesis. Indeed, these genes were up-regulated in bitter cucumber fruit (Shang et al., 2014; Zhou et al., 2016). In this study, the expression of *LaBt (Lac02g016030)* was undetected in S1174 and WM709 *Luffa* varieties (Supplementary Table S7). However, *LaBi*, *LaACT*, *La160*, *La180*, *La710*, *La890*, and *La490* were up-regulated in the bitter variety (WM709; Figure 4A; Supplementary Table S7), suggesting that they potentially participate in cucurbitacin synthesis in *Luffa*. Notably, these genes formed a Bi cluster in the synteny block between cucumber and *Luffa* (Figure 4B). However, *La170*, *La540*, *La550*, and *La560* were lacking in *Luffa* (Figure 4A), and the expression of *La510* and *La530* was undetectable in WM709 and S1174 varieties (Figure 4A), suggesting that they may not participate in cucurbitacin synthesis in *Luffa*, unlike cucumber, watermelon, and melon (Zhou et al., 2016).

Cycloartenol synthase (CAS) converts 2,3-oxidosqualene to cycloartenol through the protosteryl cation intermediate, and was the basal plant OSC from which others derived (Phillips et al., 2006). Some studies have shown that abiotic stress can up-regulate the *CAS* genes (Babiychuk et al., 2008; Shirazi et al., 2019). However, some studies reported no significant change in *CAS* expression under drought and salt stress (Basyuni et al., 2009; Nasrollahi et al., 2014). In this study, BLASTp algorithm identified *CSA* gene (*Lac05g022090*) in *Luffa* genome using *Arabidopsis* homologs for *CSA* (*AT2G07050*) as query sequences. Notably, there was no difference in *CSA* expression between S1174 and WM709 *Luffa* varieties (Supplementary Figure S5A). However, a slight up-regulation of the *CAS* gene was observed after ABA and drought treatments (Supplementary Figure S5B). Further studies are needed to determine whether CAS mediates abiotic stress response in *Luffa*.

### UGTs Regulate Cucurbitacin Glucosylation

In cucumber, UGTs regulate cucurbitacin glucosylation (Zhong et al., 2017). In this study, cucurbitacins and their glucosides were detectable and exhibited significant accumulations, with 14 up-regulated *UGTs* in WM709 (Figure 4C). Perhaps these UGTs convert cucurbitacin to cucurbitacin glucoside in *Luffa*. These results suggest that the cucurbitacin biosynthesis mechanism in *Luffa* may be similar to that in other Cucurbitaceae plants.

### Abiotic Stress Activates the Expression of Cucurbitacin Biosynthesis Genes

Cucurbitacins are biosynthesized in response to abiotic stress in Cucurbitaceae plants (Kim et al., 2020). In cucumber, exogenous ABA, drought, and cold stress up-regulated *Bi*, *ACT*, and *CYP450s* (Shang et al., 2014). However, the relationship between these genes and stress conditions has not been studied in *Luffa*. In this study, drought stress and ABA treatment up-regulated *LaBi*, *LaACT*, and *LaCYP450s* in *Luffa* (Figures 5A,B). Further analysis showed that *LaBi* promoters contain ABRE elements. Furthermore, luciferase trans-activation and yeast one-hybrid assays demonstrated that *Luffa* and *A. thaliana* AREB1 activate *LaBi* expression by directly binding to its promoter (Figures 5C,D). These results indicate that drought stress and exogenous ABA activates *Bi* expression, which promotes cucurbitacin biosynthesis, resulting in bitter fruit production in *Luffa*.

## Conclusion

**Figure 6:**
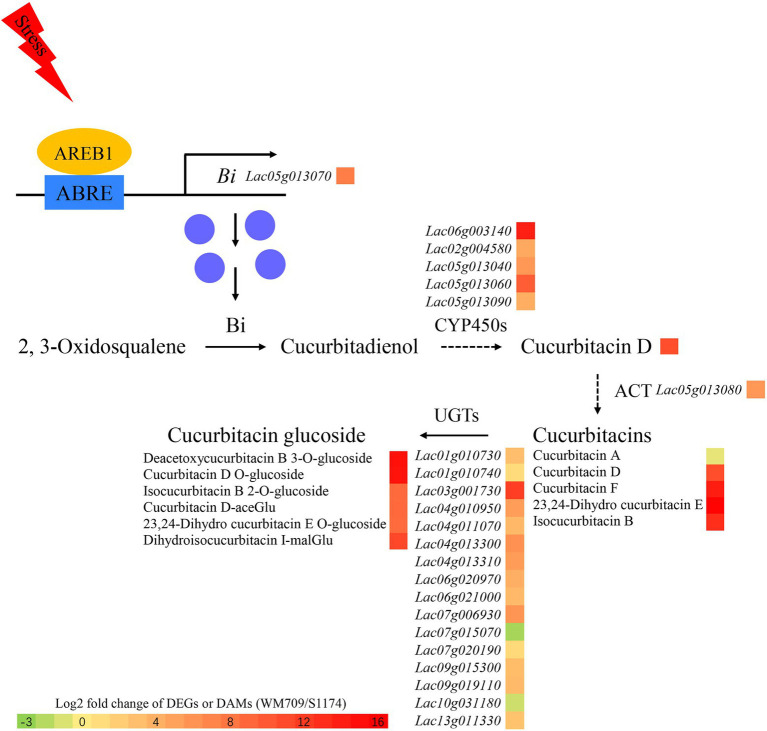
Regulatory network of predicted cucurbitacin biosynthesis. CYP450s, cytochromes P450s; ACT, acyltransferase; and UGTs, UDP-Glucosyltransferases. Green boxes indicate down-regulated transcripts and metabolites, red boxes indicate up-regulated transcripts and metabolites.

Integrated metabolome and transcriptome analysis has revealed that *Luffa* is an important medicinal plant with high nutritional value. In this study, 11 terpenoids, including cucurbitacin D, cucurbitacin F, and 23,24-Dihydro cucurbitacin E, were up-regulated in bitter *Luffa*. The cucurbitacin biosynthesis genes, *Bi*, *CYP450s*, and *ACT*, were significantly up-regulated and formed gene clusters, thereby maintaining effective biosynthesis of cucurbitacins. AREB1 binds to the Bi promoter to activate the expression of Bi in stress-exposed *Luffa* (Figure 6). These findings uncover the direct link between cucurbitacin biosynthesis and abiotic stress response and deepen the understanding of regulatory mechanisms underlying cucurbitacin biosynthesis in *Luffa*.

## Data Availability Statement

The datasets presented in this study can be found in online repositories. The names of the repository/repositories and accession number(s) can be found at: National Center for Biotechnology Information (NCBI) BioProject database under accession number PRJNA810936.

## Author Contributions

GZ, HW, and JLu conceived and designed the research. GZ, MW, CL, JLi, and XL performed the experiments. GZ, HG, and XZ analyzed the data. GZ wrote the article. All authors contributed to the article and approved the submitted version.

## Funding

This work was funded by the National Natural Science Foundation of China (31902011 and 31872093), the Laboratory of Lingnan Modern Agriculture Project (NZ2021008), the Science and Technology Program of Guangdong Province (2018B020202007, 2019A050520002, 201904020012, 2020A0505020006, 2021KJ110, and 2021A1515012500), the Science and Technology Program of Guangzhou of China (X20210201020), the Agricultural competitive industry discipline team building project of Guangdong Academy of Agricultural Sciences (202103TD and 202114TD), and Special fund for scientific innovation strategy-construction of high level Academy of Agriculture Science (R2020PY-JG004, R2018QD-035, and R2018QD-038).

## Conflict of Interest

The authors declare that the research was conducted in the absence of any commercial or financial relationships that could be construed as a potential conflict of interest.

## Publisher’s Note

All claims expressed in this article are solely those of the authors and do not necessarily represent those of their affiliated organizations, or those of the publisher, the editors and the reviewers. Any product that may be evaluated in this article, or claim that may be made by its manufacturer, is not guaranteed or endorsed by the publisher.

## Supplementary Material

The Supplementary Material for this article can be found online at: https://www.frontiersin.org/articles/10.3389/fpls.2022.886870/full#supplementary-material

Supplementary Figure S1 OPLS-DA analysis of detected metabolites. **(A)** Scores OPLA-DA plot. **(B)** R2X, R2Y, and Q2 plot.

Supplementary Figure S2 KEGG enriched pathways from the up-regulated metabolites in S1174.

Supplementary Figure S3 The correlation coefficients **(A)** and PCA **(B)** of DEGs.

Supplementary Figure S4 The number of DEGs belonging to different transcription factor families detected in S1174 and WM709.

Supplementary Figure S5 Expression profiles of LacCAS. **(A)** LacCAS read counts. **(B)** Relative expression of LacCAS after drought stress and ABA treatments.

## Footnotes

^1^ https://www.metware.cn/

^2^ http://cucurbitgenomics.org/
