# Supplementary figures and images for: Metabolome and Transcriptome Analyses of Cucurbitacin Biosynthesis in Luffa (Luffa acutangula)
Source: Front Plant Sci. 2022 Jun 7;13:886870. doi: 10.3389/fpls.2022.886870 (PMC9209774; doi:10.3389/fpls.2022.886870)

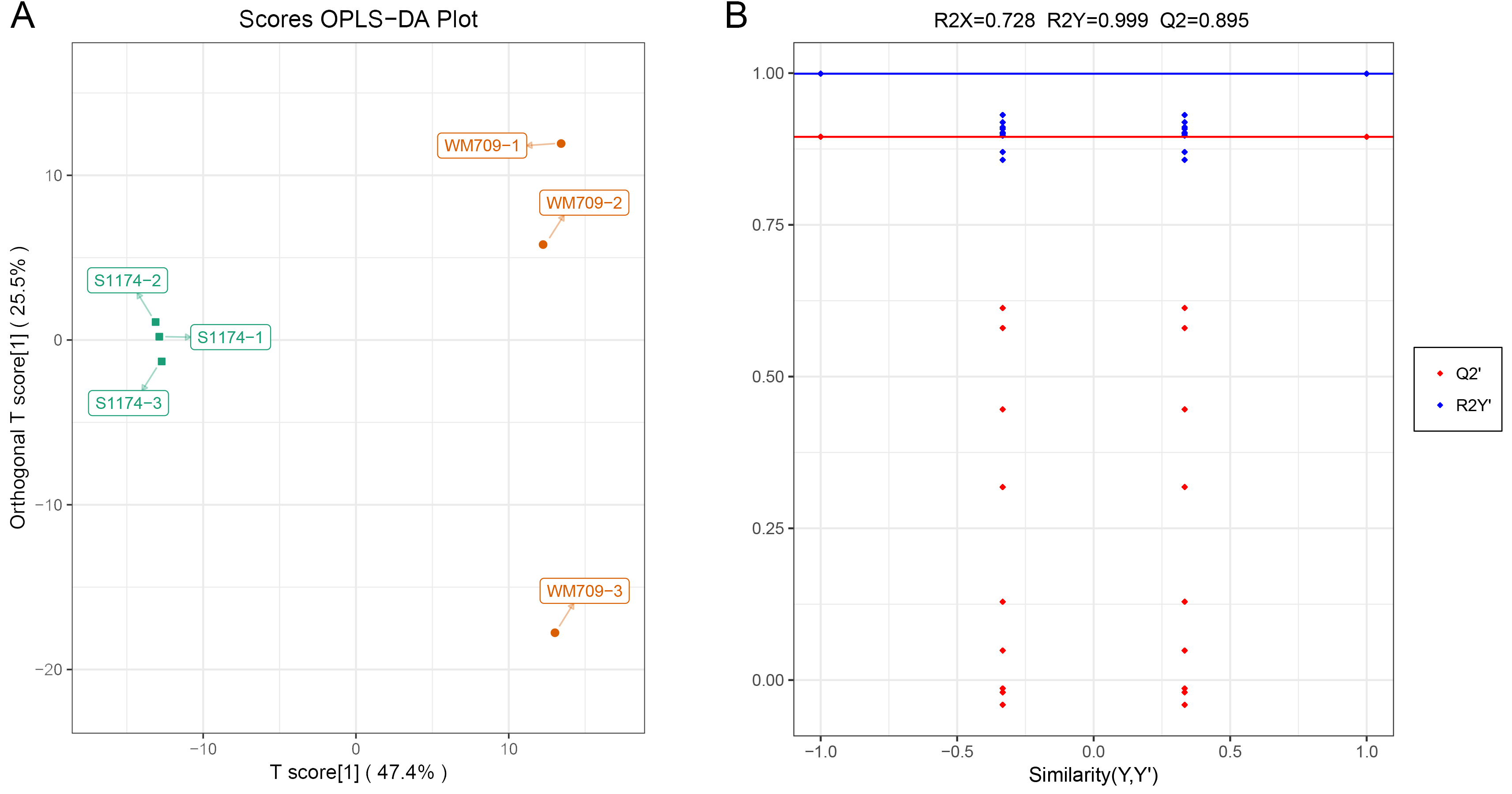

Supplement: Supplementary Figure S1 — OPLS-DA analysis of detected metabolites. (A) Scores OPLA-DA plot. (B) R2X, R2Y, and Q2 plot. [file Image_1.JPEG]

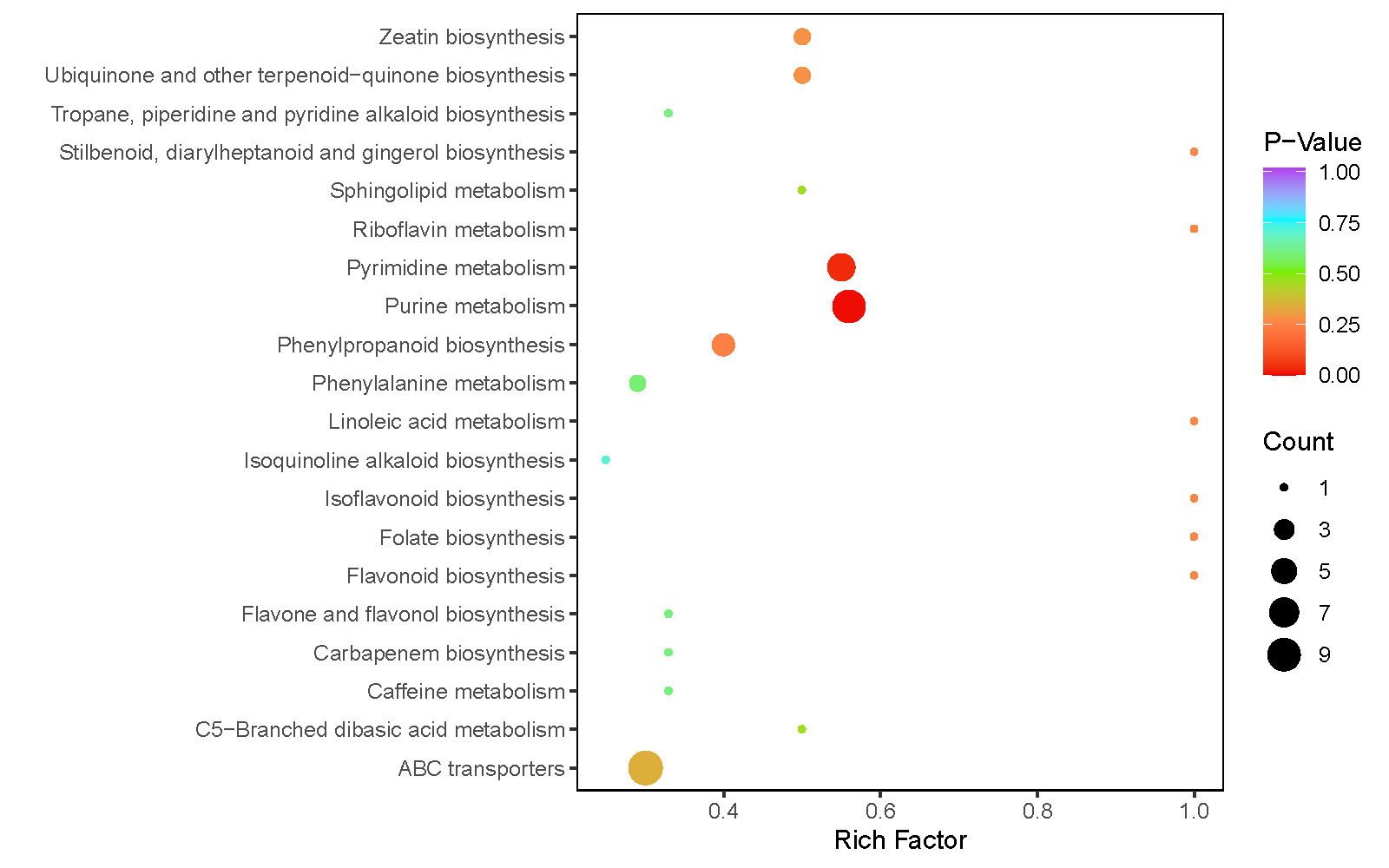

Supplement: Supplementary Figure S2 — KEGG enriched pathways from the up-regulated metabolites in S1174. [file Image_2.JPEG]

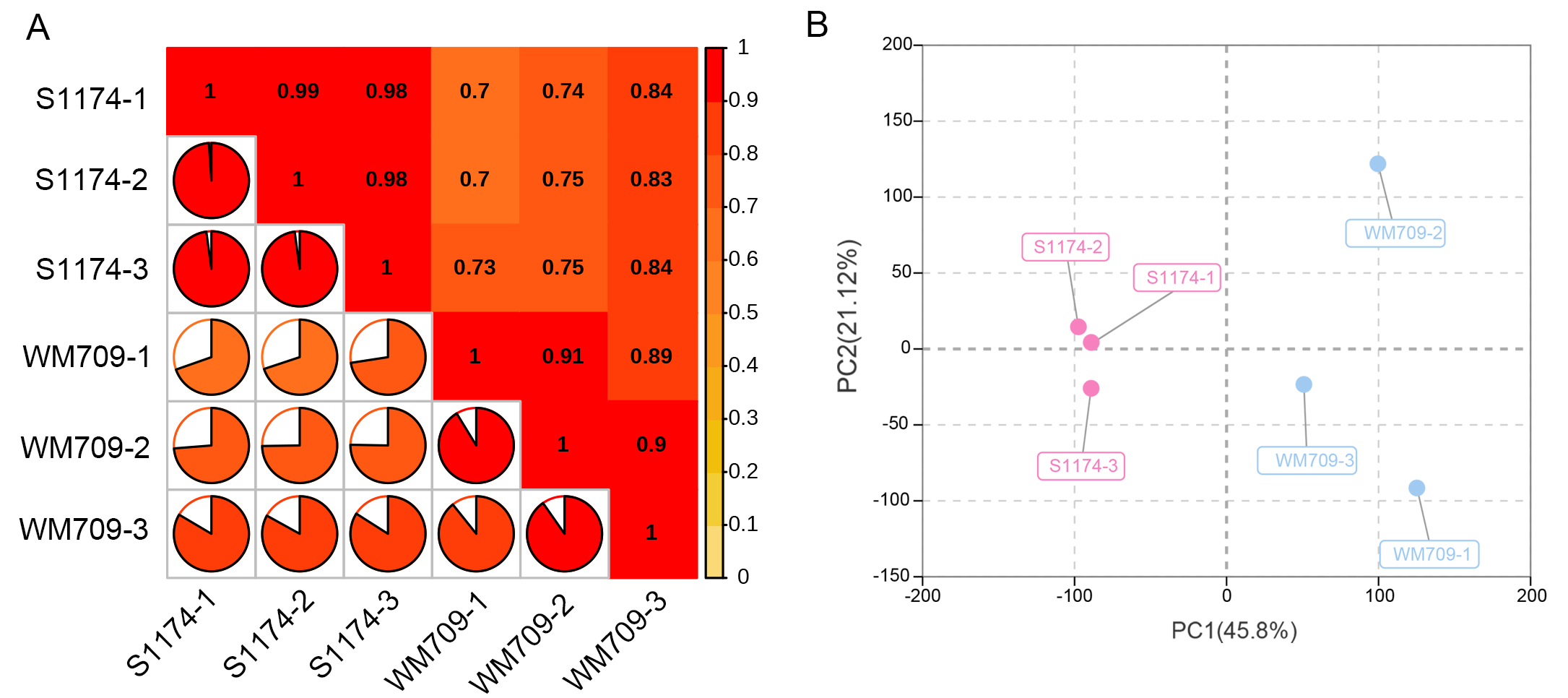

Supplement: Supplementary Figure S3 — The correlation coefficients (A) and PCA (B) of DEGs. [file Image_3.JPEG]

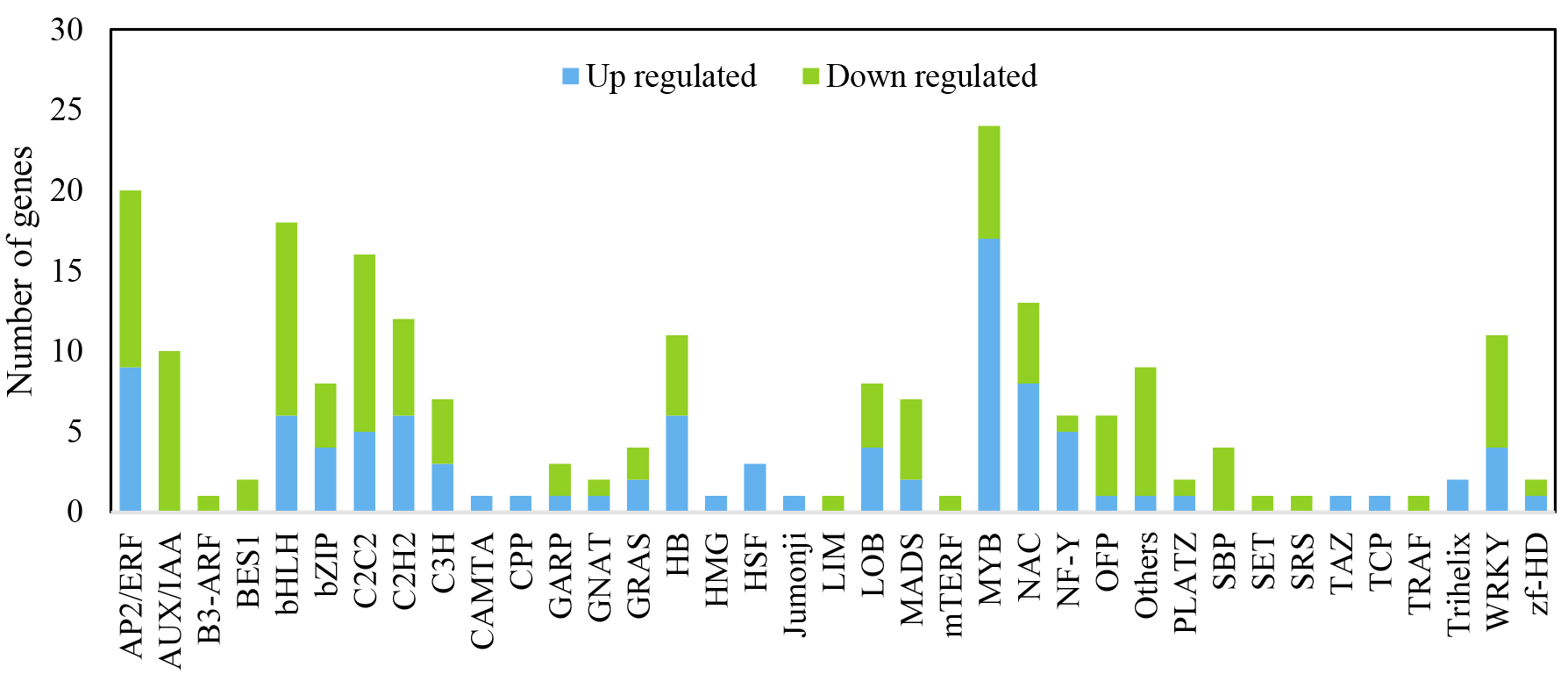

Supplement: Supplementary Figure S4 — The number of DEGs belonging to different transcription factor families detected in S1174 and WM709. [file Image_4.JPEG]

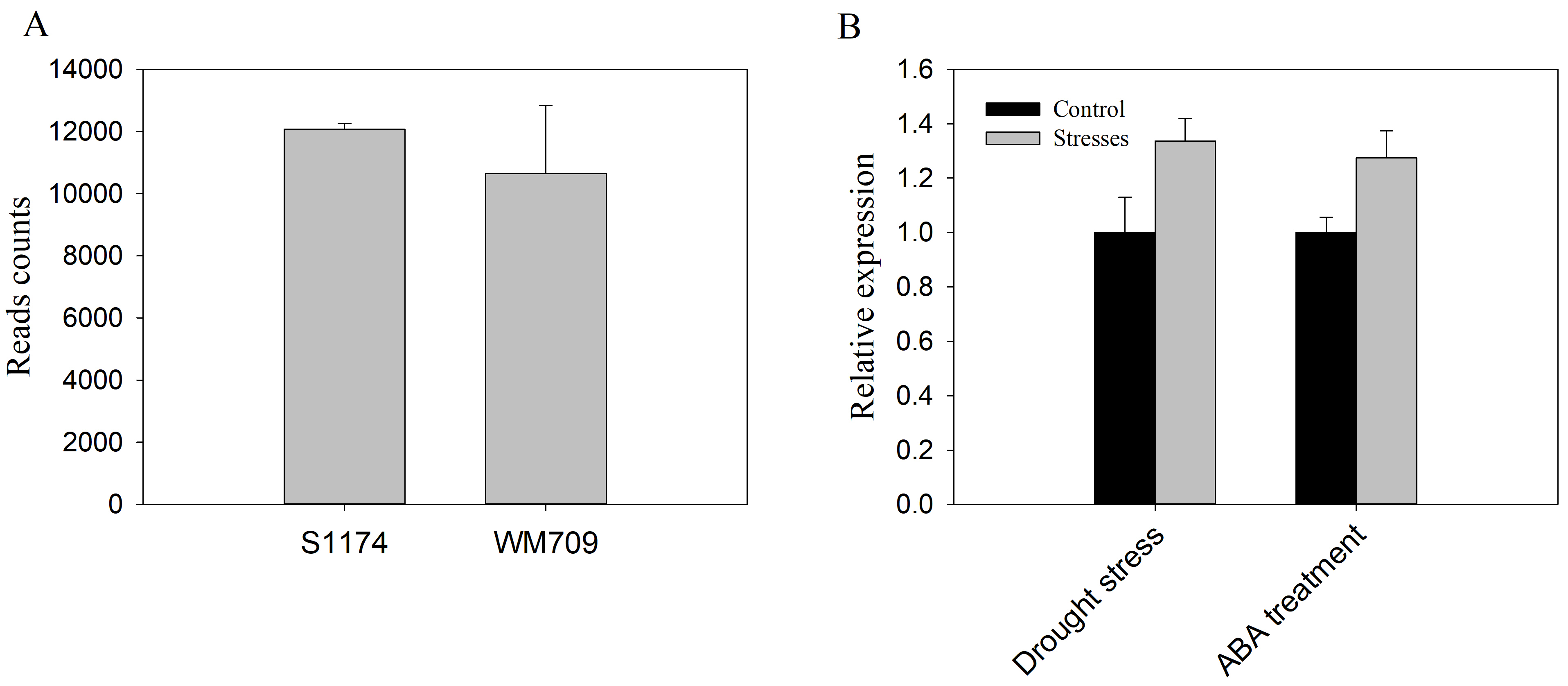

Supplement: Supplementary Figure S5 — Expression profiles of LacCAS. (A) LacCAS read counts. (B) Relative expression of LacCAS after drought stress and ABA treatments. [file Image_5.JPEG]
